# Retraction Note: The Effect of Dexmedetomidine and Esmolol on Early Postoperative Cognitive Dysfunction After Middle Ear Surgery Under Hypotensive Technique: A Comparative, Randomized, Double-blind Study

**DOI:** 10.5812/aapm.149456

**Published:** 2024-06-02

**Authors:** Mahmood-Reza Alebouyeh

**Affiliations:** 1 Iran University of Medical Sciences, Tehran, Iran

Dear Readers,

The editorial team of Anesthesiology and Pain Medicine (AAPM) is committed to maintaining the highest standards of academic integrity and ethical publishing practices. Following the publication of an Expression of Concern regarding the article titled "The Effect of Dexmedetomidine and Esmolol on Early Postoperative Cognitive Dysfunction After Middle Ear Surgery Under Hypotensive Technique: A Comparative, Randomized, Double-blind Study"(1), we have conducted a thorough investigation in response to a claim of potential misconduct (Claim #524055). 

After careful review and deliberation, we have determined that there were significant issues related to the data and content integrity of the article. Specifically, the investigation revealed discrepancies that could not be resolved satisfactorily, compromising the validity and reliability of the findings. 

The decision to retract is based on significant concerns regarding the integrity and plausibility of the data presented including Unlikely Data Distribution, Implausible Results, and Lack of Transparency (The authors failed to provide raw data upon request, which is necessary to verify the authenticity of the results)

As a result, we are retracting this article to preserve the integrity of the scientific record. This decision aligns with our commitment to ethical publishing practices and the guidelines provided by the Committee on Publication Ethics (COPE). We apologize for any inconvenience this may cause our readers and appreciate your understanding.

We remain dedicated to transparency and upholding the principles of ethical publishing. Should you have any questions or concerns regarding this matter, please do not hesitate to contact us.

Sincerely,

Head of Ethics Committee, Brieflands

On Behalf of Dr. Mahmood-Reza Alebouyeh, Editor-in-Chief
